# Psychological adjustment, quality of life, and self-perceptions of reproductive health in males with congenital adrenal hyperplasia: a systematic review

**DOI:** 10.1007/s12020-018-1723-0

**Published:** 2018-08-20

**Authors:** Elisabeth Daae, Kristin Billaud Feragen, Ingrid Nermoen, Henrik Falhammar

**Affiliations:** 1 Centre for Rare Disorders, Oslo University Hospital HF, Oslo, Norway; 2 Department of Endocrinology, Akershus University Hospital HF, Lørenskog, Norway; 3 Division of Medicine and Laboratory Sciences, Institute of Clinical Medicine, University of Oslo, Oslo, Norway; 4 Department of Molecular Medicine and Surgery, Karolinska Institutet, Karolinska University Hospital, Stockholm, Sweden; 5 Department of Endocrinology, Metabolism and Diabetes, Karolinska University Hospital, Stockholm, Sweden

**Keywords:** 21-hydroxylase deficiency, 11β-hydroxylase deficiency, Male, Psychological, Quality of life, Self-perception

## Abstract

**Purpose:**

Congenital adrenal hyperplasia (CAH) has been shown to potentially affect psychological adjustment. However, most research has focused on females, and knowledge about psychological challenges in males remains sparse. The aim of this systematic review was therefore to assess these in males with CAH.

**Methods:**

We systematically searched the OVID Medline, PsycINFO, CINAHL, and Web of Science databases, for articles published up to April 20, 2018, investigating psychological adjustment in males with CAH.

**Results:**

Eleven studies were included in the review. Three main health domains were identified: psychological and psychiatric health, quality of life (QoL), and self-perceptions of reproductive health. Some studies covered more than one health domain. Seven studies explored psychological adjustment and/or the presence of psychiatric symptoms or disorders. Results indicated that males with CAH had more problems related to internalizing behaviors (negative behaviors directed toward the self) and more negative emotionality compared to reference groups. Six studies examined QoL, five of them reporting reduced QoL compared to reference groups. Three studies explored the impact of fertility and sexual health issues on psychological health with varying results from impaired to normal sexual well-being.

**Conclusions:**

CAH seems to have an impact on males' psychological health. However, the number of identified studies was limited, included few participants, and revealed divergent findings, demonstrating the need for larger studies and highlighting a number of methodological challenges that should be addressed by future research.

## Introduction

Congenital adrenal hyperplasia (CAH) is a spectrum of genetic disorders causing deficiencies in the steroidogenic enzymes in the adrenal cortex [1]. Of all cases, 95–99% are due to 21-hydroxylase deficiency (21OHD) with defective 21-hydroxylase enzyme, resulting in impaired production of cortisol and varied degree aldosterone, in addition to increased synthesis of adrenocorticotropic hormone (ACTH), steroid precursors, and adrenal androgens [1–5]. 11β-hydroxylase deficiency (11βOHD) is the second most common variant of CAH, which in contrast to 21OHD demonstrate elevated mineralocorticoid precursors and mild-to-moderate hypertension [6].

Clinically 21OHD is divided into classic CAH, which includes the salt-wasting (SW) and simple virilizing forms (SV), and non-classic (NCAH) form [1, 7, 8]. Girls with classic CAH are born with virilized external genitalia while boys with classic CAH have no overt symptoms of adrenal hyperandrogenism at birth. However, since SW entails complete lack of cortisol and aldosterone, the infant boy or girl with SW will die of a salt-losing crisis within the first few weeks if not diagnosed and properly treated [3, 9]. Before the introduction of neonatal screening for CAH [10], the boys with SV were diagnosed at 3–28 years of age (mostly at the lower end of the range) with signs and symptoms of androgen excess (including acne) or during family screening [11], or even later due to adrenal incidentalomas [12]. NCAH does not have overt cortisol or aldosterone deficiency but has manifestations of hyperandrogenism in women typically presenting later in childhood or in early adulthood, while being usually asymptomatic in men [13, 14].

Individuals with classic CAH require lifelong treatment with glucocorticoid, and often mineralocorticoids as well [2, 3, 15], to prevent adrenal crisis with potential fatal outcomes [16, 17], and to normalize the adrenal androgens. However, glucocorticoid excess can result in growth suppression in children, obesity, diabetes, hypertension, increased cardiovascular risk, decreased bone mineral density, fractures, and adverse psychological effects [5, 11, 18–23]. Whereas the routine and centralized follow-up of females allows for a close monitoring and supervision of potential physical and psychological consequences, the follow-up of adult males with CAH remains less systematic and lost to follow-up is common.

Testicular rest tumors (TARTs) in males with CAH may impact fertility [23–28], and psychological and emotional factors may also impact sexual health as well [29]. Fertility has been reported to vary from normal to severely impaired in males with CAH when compared to national data or control groups [23, 25, 27, 30–32].

An association between CAH and mood disorders was described several decades ago [33], and has been replicated in more recent research [17, 34], indicating that glucocorticoids given as a pharmacological treatment, and not only as a substitute, may cause psychological and psychiatric symptoms like anxiety, insomnia, behavior disturbances, mood disorders, and psychotic disorders [35]. There is also evidence of androgens' influence on emotional reactivity, brain structure, and function, suggesting that young people with hyperandrogenism, such as in CAH, could be at risk of developing psychological symptoms or psychiatric disorders [36]. Psychosocial adaptation difficulties due to a lack of sexual well-being and fertility issues could also be expected in males with CAH [37].

Psychological research on females with CAH has investigated the impact of androgens on gender typical play [38], sex-typed toy preference [39, 40], gender identity [41, 42], gendered occupational interest [43–45], sexual behavior [45–47], levels of aggression [48], and spatial abilities [49]. Females with classic 21OHD have also served as human models for the study of early androgen exposure effects on the developing brain [50, 51]. While knowledge about females with CAH is important and needed, there is a definite lack of literature exploring the potential psychological consequences of CAH in males, probably explained by the assumption that males with CAH are exposed to normal prenatal androgen levels [52].

Additionally, the chronicity of CAH entails daily medication, close monitoring by parents during childhood, and when needed also by medical health professionals, experiences that in spite of the positive medical benefit of follow-up, have been shown to restrict physical and social development in children with chronic disorders in general [53]. Whereas some studies have focused on the medical aspects of the condition in males, few studies have explored how CAH could potentially affect these patients' psychological adjustment and quality of life (QoL) [54]. Consequently, in order to examine whether males with CAH present distinct psychological challenges that would need to be addressed clinically, an updated summary of the literature is needed.

The aim of the current systematic review was therefore to examine and summarize the existing psychological research on males with CAH, in order to identify potential challenges in psychological and sexual health and QoL that could be associated with consequences of the diagnosis and its treatment.

## Materials and methods

### Inclusion and exclusion criteria

A systematic review of the psychological literature of men with CAH was performed, following the PRISMA statement [55]. All original peer-reviewed articles, published before April 20, 2018 and investigating psychological adjustment in males with CAH were included. Studies using quantitative, qualitative, and mixed methods were all considered. No age restrictions were imposed, and all methods of measurement (self-report, parent-report, and third-party reports) were included. Unpublished dissertations, case reports, review articles, editorial, and meeting abstracts were excluded. Further exclusion criteria were: (a) studies with mixed samples that did not present results separately for males and females, (b) studies with females only, or (c) studies focusing on disorders of sex development as a group, with results not being displayed separately for CAH.

### Search strategy

The OVID Medline, PsycINFO, CINAHL, and Web of Science databases were systematically searched, using the following search terms: congenital adrenal hyperplasia, CAH, adrenogenital syndrome, 21-hydroxylase deficiency, men, boy, male, sex*, psychological, psychological adaptation, distress, puberty, stress, affective, quality of life, qol, emotional, psychosocial, mental disorder, social stigma, lived experience, resilie*, satisfaction, empower*, attitude*, coping*, knowledge, information, network, participat*, isolation, relation*, stigma*, screening, and social adaptation. The Boolean operators OR were used between search terms within diagnostic, gender, and psychological search terms, while the operator AND was used between the three categories. No filters were used in the search strategy. The articles were initially screened by title for relevance and then by abstract and subsequent potentially relevant articles were full-text reviewed. The reference lists of the retrieved full-text articles were scanned to identify any additional relevant articles.

### Assessment of methodological quality

**Fig. 1:**
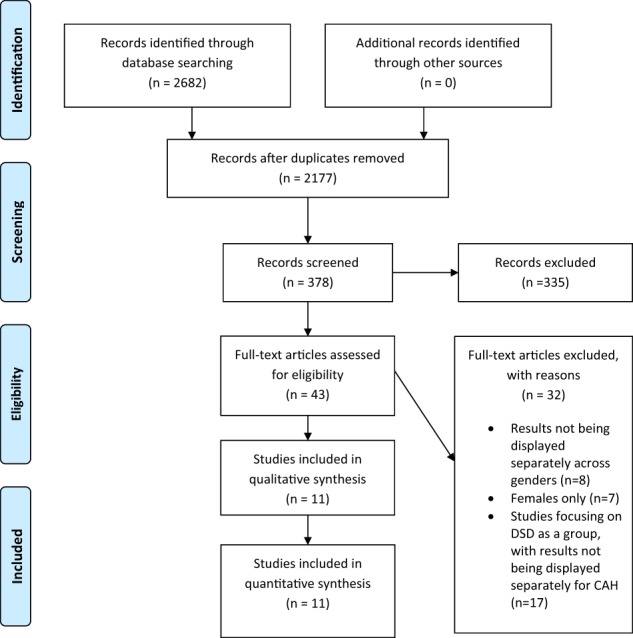
Illustrating the procedure for article inclusion and exclusion in a systematic review of psychological adjustment, quality of life, and self-perceptions of reproductive health in males with congenital adrenal hyperplasia

The systematic search initially yielded 2682 articles and after removal of duplicates, 2177 records were screened for eligibility (Fig. 1). By examining all titles, 378 studies were identified. Abstracts were assessed according to the agreed criteria, leading to the exclusion of 335 papers. Hence, a total of 43 articles were selected for full-text reading. Included and excluded articles were agreed on together with reference to the criteria described by all authors, and 11 studies were finally included [5, 29, 36, 37, 54, 56–61].

### Data extraction

Data were collected regarding the included authors, country, measures, gender, methods, design, setting/context, and results/findings.

## Results

An overview of the 11 articles can be found in Table 1. All articles were quantitative. The measures and domains investigated in each paper are described in Table 2. Three main themes were identified: (1) psychological and psychiatric health, (2) QoL, (3) perceptions of reproductive health: fertility and sexual function.

**Table 1:** Overview and details about the included articles of the present systematic review

Reference	Sample size	Age range (years)	CAH type	Informants	Recruitment site	Comparison group	Design
Arlt et al. [5]	65 males	18–69	Majority 21OHD	Patients	UK	Health Survey for England data and reference cohorts	Quantitative
Berenbaum et al. [61]	42 males	3–19 11–31	21OHD Sample 1: SW (84%) Sample 2: SW (67%)	Patients and parents	USA	Healthy controls Male relatives Population average	Quantitative
Dudzinska et al. [37]	20 males	18–49	21OHD SW (*n* = 14) SV (*n* = 6)	Patients	Germany	Healthy controls Norwegian norms	Quantitative
Falhammar et al.^a^ [76]	253 males	0.5–80	21OHD SW (*n* = 105) SV (*n* = 76) NCAH (*n* = 19)	Patients from longitudinal nationwide population-based registers	Sweden	Register-based male controls, matched by birth year, sex, and place of birth (*n* = 25,300)	Quantitative
Falhammar, Nyström, and Thorén [29]	30 males	19–67	21OHD Classic (*n* = 28) NCAH (*n* = 2)	Patients	Sweden	Sex- and age-matched controls	Quantitative
Gilban et al. [54]	6 males	5–17.9	21OHD SW (*n* = 4) SV (*n* = 2)	Parents and patients	Brazil	Healthy controls	Quantitative
Idris et al. [58]	20 males	6–18	Clinical and biochemical characteristics	Parents	Malaysia	Siblings American norms	Quantitative
Jacobs et al. [56]	5 males	16–55	21OHD (*n* = 2) 11βOHD (*n* = 3)	Patients	USA	None	Quantitative
Mueller et al. [36]	33 males	8–18	Majority 21OHD SW (*n* = 20) SV (*n* = 11)	Parents and patients	USA	Population estimates Other chronic disorders	Quantitative
Reisch et al. [60]	36 males	18–65	Classic 21OHD	Patients	Germany	Sex- and age match controls and patients with partial androgen insensitivity syndrome	Quantitative
Strandquist et al.^a^ [57]	253 males	0.5–80	21OHD SW (*n* = 105) SV (*n* = 76) NCAH (*n* = 19)	Longitudinal nationwide population-based registers	Sweden	Register-based male controls, matched by birth year, sex, and place of birth (*n* = 25,300)	Quantitative

*CAH* congenital adrenal hyperplasia, *21OHD* 21-hydroxylase deficiency, *11βOHD* 11β-hydroxylase deficiency, *SW* salt-wasting, *SV* simple virilizing

^a^The same patient cohort and controls were used but different aspects were investigated in the two studies

**Table 2:** Overview of included articles studying males with congenital adrenal hyperplasia with measures and investigated domains

Reference	Measures	Psych	QoL	SPRH	Summary of findings
Arlt et al. [5]	Short Form Health Survey (SF-36) Hospital Anxiety and Depression Scale (HADS) The International Index of Erectile Function Questionnaire (IIEF-5) CAH-Well-Being Questionnaire	x	x	x	Reduced QoL Increased anxiety scores Erectile dysfunction in 41%
Berenbaum et al. [61]	Child Behavior Checklist (CBCL) Self-Image Questionnaire for Young Adolescents (SIQYA) Multidimensional Personality Questionnaire (MPQ)	x			Higher on negative emotionality
Dudzinska et al. [37]	Short Form Health Survey (SF-36) Giessen Subjective Complaints List (GBB-24) Hospital Anxiety and Depression Scale (HADS) Brief Sexual Function Inventory (BSFI)	x	x	x	Reduced QoL Higher depression and anxiety scores Some sexual problems
Falhammar et al. [59]	Psychiatric diagnoses according to ICD-8, -9, and -10	x			Increase of psychological disorders and alcohol misuse
Falhammar, Nyström, and Thorén [29]	McCoy modified Psychological Well-being Index (PGWB)		x	x	Sexual satisfaction and QoL similar to controls Less sexually active
Gilban et al. [54]	Child Health Questionnaire - Parent Form (CHQ-PF50) Pediatric Quality of Life Inventory (PedsQoL 4.0)		x		Impairment of HRQoL
Idris et al. [58]	Child Behavior Checklist (CBCL)	x			Adverse psychological adjustment
Jacobs et al. [56]	Tension Scales of the Profile of Mood States (POMS)	x			Adrenal suppressive therapy effective on anxiety disorders
Mueller et al. [36]	Schedule for Affective Disorders and Schizophrenia for School Aged Children (KSADS-PL)	x			Increased rates of anxiety disorders & disruptive behavioral disorders
Reisch et al. [60]	Short Form Health Survey (SF-36) Giessen Subjective Complaints List (GBB-24) Hospital Anxiety and Depression Scale (HADS)		x		Mildly impaired QoL
Strandquist et al. [57]	Proxies for QOL		x		QoL parameters differed significantly from matched controls

*Psych* psychological and psychiatric, *QoL* quality of life, *SPRH* self-perceptions of reproductive health

### Psychological and psychiatric health

Seven studies addressed psychological and psychiatric health [5, 36, 37, 56, 58, 59, 61], two of these investigating behavioral problems [58, 61]. The first study by Idris et al. [58] was based on a sample of 20 Malaysian males with CAH, categorized as SW or SV, aged 6–18 years. Results were compared with unaffected siblings, using norms based on American samples. Age and sex did not match between patients and controls, therefore relatives of male and female patients were combined to form male and female control groups. Males with CAH did not differ from controls on problems with externalizing behavior (negative and problematic behaviors that are directed toward the external environment). However, in the clinical sample, findings revealed scores within the clinical range on problems with internalizing behavior (negative and problematic behaviors that are directed toward the self), indicating difficulties related to anxiety and depression, in addition to withdrawn behaviors and somatic complaints. Berenbaum et al. [61] included two samples of males with CAH (21OHD). In the first sample (3–19 years), males with CAH did not differ from unaffected male relatives or population averages on measures of behavior or self-image. In the second sample (11–31 years), males had higher scores than controls on negative emotionality.

Two other studies, investigating levels of anxiety and depression, reported increased anxiety scores in British males aged 18–69 years with CAH (majority of 21OHD) [5], and higher depression and anxiety scores in German males with CAH (21OHD, 14 SW and 6 SV) aged 18–49 years [37], when compared to healthy controls. A follow-up after 2 years in the latter study showed no longitudinal changes in psychological symptoms.

Mueller et al. [36] investigated 33 American males with CAH (mainly 21OHD), aged 8–18 years. Relative to the population estimate, the findings indicated increased rates of anxiety disorders, disruptive behavioral disorders, in addition to attention difficulties and hyperactivity disorders (ADHD). When they compared their results with other chronic disorders (such as diabetes or uncomplicated epilepsy), more elevated rates of ADHD were found in the sample with CAH. Differences were also found between subtypes of CAH: 18% of males with SV were found to have mood disorders, compared to none of the males with SW. When comparing figures across gender, 51–56% of the males were found to have a psychiatric disorder, compared to 33% of the females.

Jacobs et al. [56] based their study on five males with NCAH (21OHD and 11βOHD), aged 16–55 years, who had been diagnosed with refractory anxiety disorders. The patients were initiated on ketoconazole (as an adrenal suppressive therapy) and then glucocorticoids if further adrenal suppressive therapy was required. Anxiety was measured before and after treatment. A reduction in dehydroepiandrosterone sulfate (DHEAS) was associated with lower anxiety scores, and Tension Scale of the Profile of Mood States (POMS Tension) scores decreased by 55% in the total sample (males and females), suggesting that NCAH can contribute to anxiety disorders, while also demonstrating the potential effectiveness of pharmacological therapy.

Falhammar et al. [59] examined psychiatric morbidity in 253 Swedish males with CAH (21OHD), and compared with 25,300 control males, matched by age and place of birth. The study gathered information from the National Patient Registry, which contained ICD diagnoses of both inpatient and outpatient care, and the cause of death registry. Analyses revealed that the occurrence of psychiatric disorders and alcohol misuse was increased by 50% in adult males with CAH when compared to controls. Males with SW showed a significantly higher frequency of personality disorders and alcohol misuse, whereas drug abuse was increased in males with SV. Suicidality was almost doubled in males with CAH compared to controls, in addition to increased risks of anxiety symptoms. Males born before the introduction of neonatal CAH screening (1986) had higher rates of suicidality and psychiatric disorders, and a tendency toward a higher frequency of personality disorders, when compared to controls. Patients born after the introduction of neonatal screening had a higher prevalence of psychotic disorders, whereas findings were less clear-cut regarding substance abuse.

In summary, studies investigating psychological and/or psychiatric symptoms reported significant problems in males with CAH compared to relevant reference groups.

### Quality of life

A total of six studies included measures of QoL [5, 29, 37, 54, 57, 60]. Gilban et al. [54] included six young males with CAH (21OHD), aged 5–17.9 years, using both parent and self-reports. Self-reports of health-related QoL suggested lower scores on the physical, academic, and psychosocial dimensions compared to the control group. Similarly, parent reports showed lower scores on all dimensions when compared to healthy controls. However, the study raises some methodological considerations. Two patients with 46,XX karyotype opting for male social sex were included in the sample, in addition to three 46,XX patients that had been assigned as boys at birth. It is unclear whether these patients were included in the male sample. Given the study’s small sample size, the results should therefore be interpreted with caution.

Falhammar, Nyström and Thorén [29] included 30 adult males with 21OHD, comparing results with 32 age-matched male controls. Mean QoL scores were not statistically different between the two groups. In contrast, two other studies with samples of males ranging from 6 to 65, reported reduced QoL in their samples when compared with reference data or healthy controls [5, 37] while the third study found mildly impaired QoL [60]. This study also reported reduced health-related QoL compared to females with the same condition, but higher QoL compared to males with primary adrenal insufficiency, a patient group that also receives glucocorticoid supplementation [60].

Two studies reported demographic factors that could be seen as parameters of QoL. The first study, Strandqvist et al. [57], consisted of 253 Swedish males with CAH (SW, SV, and NCAH) and 25,300 age- and place of birth-matched controls. This cohort of CAH males and controls was the same as in Falhammar et al. [59], but other parameters were studied. The outcome data were extracted from several national registries. Males with the less severe genotype were more likely to have an academic education than controls (OR 1.8). In contrast, males with CAH also had more disability pension (OR 1.5) and sick leave (OR 1.7), but were more likely to have been employed for more than 7 years (OR 3.1). Nonetheless, family income did not show significant differences with controls. The second study, Falhammar, Nyström and Thoren [29], indicated that the majority of males in both groups (21OHD and controls) had full-time engagements such as work or studies (86% and 88%, respectively). Furthermore, differences were found in how many who were students (3% vs. 25%), and in the number of individuals engaged in blue-collar work (57% vs. 33%).

Arlt et al's study [5] consisted of 65 males with CAH (mainly 21OHD) filling out SF-36. The results were compared to sex- and age-matched controls. Subjective health status was significantly impaired across all eight SF-36 domains. Dudzinska et al. [37] found no significant changes in *z*-scores during the 2-year study period in the adult CAH cohort.

To summarize, five of six studies investigating QoL, and including the majority of patients, reported reduced QoL in men with CAH compared to the reference population.

### Perceptions of reproductive health: fertility and sexual function

A total of three studies investigated males' perceptions of sexual and reproductive health [5, 29, 37]. Dudzinska et al. [37] included 20 male patients, and were the first to describe an impaired sexual well-being in males with CAH (21OHD). The study examined clinical, genetic, biochemical, and hormonal parameters in German males, and results were compared with normative data from Norway. Results indicated impairments in the dimensions “sexual drive”, “erections”, and “ejaculations”, whereas the dimensions “problem assessment” and “overall satisfaction” revealed scores within the normal range. In the study by Falhammar, Nyström and Thoren [29], sexual satisfaction was similar to controls, even though fewer males with CAH (21OHD) reported to be sexually active. Arlt et al. [5] reported erectile dysfunction in 41% of males with CAH (mainly 21OHD). The CAH Wellbeing Questionnaire also revealed that 24% of the males were concerned about the diagnosis' impact on their long-term health. Furthermore, a significant number of patients were concerned about their height (35%), weight (46%), fertility issues (26%), and 38% were unhappy about their sex life.

## Discussion

The literature on CAH has mainly focused on gender issues and psychosocial adjustment in females [42, 45, 62, 63]. Both genders face some of the same challenges, such as living with a rare chronic disease, the risk of glucocorticoid over- and undertreament, with corresponding disturbances of weight and growth development, school absences due to frequent medical procedures, possible androgen effects on brain development and behavior, risks for impaired reproductive health, and/or potential psychosocial and emotional issues that could be associated to the condition and its treatment. Some challenges may also be specific to males, such as the potential development of TARTs, their possible impact on self-perceptions of reproductive health and the risk for hypogonadotropic hypogonadism secondary to poor control of CAH. No reviews have previously addressed the specific psychological and emotional challenges that may be found in males with CAH. This systematic review therefore examined the literature pertaining to psychological health in males with CAH, and identified three main domains: “Psychological and psychiatric health”, “QoL”, and “Self-perceptions of reproductive health: Fertility and Sexual function”.

Studies investigating psychological adjustment and/or the presence of psychiatric symptoms or disorders, indicated more emotional problems in males with CAH than in reference groups. The lack of longitudinal data sets examining psychological adjustment and/or the presence of psychiatric symptoms or disorders, except for one study investigating the development of anxiety over a period of 2 years [37], impede conclusions about whether and how such problems may fluctuate over time in males with CAH. In addition, there is a lack of knowledge about the nature of contributing factors affecting the development of emotional difficulties in males with CAH. Investigations of the effect of adrenal suppressive therapy on anxiety disorders have demonstrated that pharmacological therapy could contribute in reducing anxiety levels [56]. Another potential factor of interest was illustrated by Falhammar et al. [59], who included males before and after neonatal screening was introduced. Reduced levels of suicidality and psychiatric disorders were found in males born after the introduction of neonatal screening, hence providing evidence of androgens' influence on emotional reactivity, brain structure and function, a potential co-factor in the development of psychiatric and/or psychological disorders [36]. Thus, further research is needed to investigate psychological and emotional risk in males with CAH, in order to have a better understanding of underlying or precipitating risk factors.

Conflicting findings reported in the present review may partially be explained by methodological disparity, such as a wide variation in designs, samples, and outcome measures, in addition to a potential variation in subgroups of CAH. Other explaining factors could be related to the multifaceted and complex nature of psychological adjustment and well-being. QoL is a multidimensional concept, as is psychological adjustment, involving physical, social, and cultural aspects [64]. Findings may therefore be expected to vary according to whether the design has been controlled for the variation of such confounding factors. Given the methodological disparity in studies within this review, more research is required.

Five studies not included in this review, due to results not being presented separately across genders, also concluded with health-related Qol being affected in people living with CAH [65–69]. Halper et al. [65] report no differences in scores between males and females, but findings were not presented separately, and this paper was therefore not included. The sample consisted of 45 children with CAH. Among those, 32 filled out self-reported QoL (PedsQL Generic Core Scale and PedsQl Fatigue Scale), and 44 parents completed a parent proxy-report form. The children did not report lower overall QoL except for the physical health subscale. The authors assume it might be explained by the children being on hydrocortisone, which has been found to have a less negative impact on QoL in adults than other glucocorticoids [67]. Yau et al. [66] recruited 33 parents and their children with CAH, who also completed measures of QoL (PedsQL 4.0 generic core scales). Scores were compared to children with hypothyroidism. Children with CAH reported lower scores in the school domain, and more psychosocial problems. No differences were found in parent reports. Han et al. [67] investigated QoL (Short Form Health Survey) in 151 adults with CAH aged 18–69 years, and also recording glucocorticoid regimen, anthropometric, and metabolic measures. Results indicated lower QoL in adult patients with CAH using prednisolone and dexamethasone compared to those taking only hydrocortisone. Further, Nermoen et al. [68] conducted a QoL survey in Norway, studying patients with classic CAH. The results showed that subjective health status and working ability were impaired in males and females. In contrast, a Finnish study [69], including men and women with CAH, showed a better health-related QoL than the general Finnish population. Yau et al. [66] found no differences in overall or specific health-related QoL between males and females, but again results were not presented separately. Halper et al. [65] did not find any differences in QoL scores between sex and CAH subtypes. The results from these studies confirm that males with CAH may not have less psychological problems than females with CAH, highlighting the need for future research on this specific population.

The pathophysiology leading to fertility problems in males is not fully understood [70], but seems to be related to the prevalence of TARTs and inadequate medical control of the condition, either due to undertreatment or non-compliance to treatment [70]. Whereas several studies have investigated fertility and reproductive function in males with CAH [23, 25, 27, 28, 30, 31], the present review only identified three papers that had explored patients' own satisfaction with reproductive health [5, 29, 37].

Males with CAH can experience precocious puberty [71], which can be an unpleasant experience for a child. Further, poor disease control may be associated with reduced sexual drive, linking the treatment of CAH directly to sexual well-being [37]. Risks for obesity and shorter height stature [72] as a consequence of glucocorticoid treatment [73] may also potentially affect psychosexual adjustment and reproductive health in males with CAH. The present review demonstrates the need for further research exploring males with CAH's subjective satisfaction with reproductive health and sexual function, in order to better determine this factor’s possible association with psychological adjustment and QoL.

The present systematic review confirms the need for more systematic routine follow-ups of males with CAH, in order to identify those at risk for developing psychological difficulties and provide adequate support and help [74]. Multidisciplinary teams are essential, including specialists in endocrinology, and genetics, in addition to clinical psychologists and psychosocial services [74], in females as well as in males. Systematic follow-up should examine the patient's experience of the medical condition and its consequences, and include an opportunity to discuss treatment opinions. Furthermore, treatment issues, questions related to providing information about the diagnosis to others, and building up necessary knowledge about the condition should be implemented in patient care [75]. Information provided to parents of young children gradually needs to be transferred to the young people themselves, in order to build empowerment, and equip young patients with the skills and knowledge necessary to manage their own healthcare. In order to strengthen adolescent transition programs, building an infrastructure that includes training and education of healthcare professionals are also recommended and needed [73]. General practitioners and other physicians who treat adult patients with CAH should also ensure that these are followed-up by an endocrinologist.

The present systematic review has highlighted a number of methodological challenges and limitations that prevented us from performing a meta-analysis. First, the number of instruments used to measure similar aspects of psychological health and QoL was apparent, and complicates the comparison of findings across studies. Further, studies using the same measures still build upon different designs and samples, again complicating comparisons. Another methodological challenge was that several studies included both genders in the sample, without presenting findings separately, impeding the investigation of gender-specific difficulties in their patient samples. These studies were therefore not included, suggesting cautiousness in conclusions regarding the present findings. Third, as could be anticipated, relatively few studies drew upon large samples. Eight of the 11 included studies had less than 50 participants with CAH, one study had 65, while only two studies had 253 males with CAH. Another methodological challenge was related to participants representing a broad age range. This is understandable given the rarity of the diagnosis, but impacts on the validity and generalizability of findings, a challenge that could be addressed by multicenter and international studies. Furthermore, two of the studies used norms from another country than the one they conducted in the study refs. [37, 58], a methodological issue that may have implications for the interpretation of the results. A positive finding was that most studies had control groups, and findings could therefore more easily be compared to relevant reference groups.

An additional consideration in investigating the impact of medical diagnoses on psychological health is whether to use generic on condition-specific measures. All studies included in the present review were based on generic outcome measures, except for the CAH Wellbeing Questionnaire used by Arlt et al. [5]. The advantage of using generic measures of adjustment and health is enhanced prospects of comparisons between patient groups and controls. On the other hand, complementary condition-specific outcome measures that capture distinct psychological aspects and challenges associated with a specific diagnosis may be needed to fully capture the complexities of the condition itself. However, the development and evaluation of new outcome measures is a lengthy and challenging process, and several existing condition-specific measures are therefore not validated and seldom used across studies. Nevertheless, efforts should be made to help the development of condition-specific measures in future research, and also for researchers to agree on using the same measures when investigating similar domains of health

Surprisingly, the current systematic review did not include any studies based on qualitative research, in spite of this methodological approach being well suited to the rare nature of CAH, and the present lack of knowledge about the psychological impact of the condition in males.

In conclusion, the small number of studies identified and included in the present systematic review illustrates the need for further research on the psychological impact of CAH on males. Males with CAH appeared to display psychological and psychiatric symptoms, impaired QoL, in addition to reduced satisfaction with reproductive health and sexual function. Larger samples are needed in future research, which can only be achieved through national and international multicenter collaborations. Future studies could also use qualitative methodologies, for gaining an in-depth understanding of the patient perspective, identify areas of particular relevance to clinical care, and hence advance our knowledge of specific challenges experienced by males with CAH. One step further, which would be well suited to the multidimensionality and complexity of adjustment to CAH, would be to combine quantitative and qualitative approaches, in the form of mixed methods studies. Long-term follow-up of males with CAH, including elements of psychological screening, prevention, and treatment is important.
